# U-Net combined with multi-scale attention mechanism for liver segmentation in CT images

**DOI:** 10.1186/s12911-021-01649-w

**Published:** 2021-10-15

**Authors:** Jiawei Wu, Shengqiang Zhou, Songlin Zuo, Yiyin Chen, Weiqin Sun, Jiang Luo, Jiantuan Duan, Hui Wang, Deguang Wang

**Affiliations:** 1 School of Medical Imaging, Xuzhou Medical University, Xuzhou, China; 2 School of Economics and Finance, Xi’an Jiaotong University, Xi’an, China; 3 School of the First Clinical Medical, Xuzhou Medical University, Xuzhou, China; 4 Jiangsu Union Technical Institute, Xuzhou, China

**Keywords:** Deep learning, Attention mechanism, Multi-scale, Liver segmentation, CT images

## Abstract

The liver is an important organ that undertakes the metabolic function of the human body. Liver cancer has become one of the cancers with the highest mortality. In clinic, it is an important work to extract the liver region accurately before the diagnosis and treatment of liver lesions. However, manual liver segmentation is a time-consuming and boring process. Not only that, but the segmentation results usually varies from person to person due to different work experience. In order to assist in clinical automatic liver segmentation, this paper proposes a U-shaped network with multi-scale attention mechanism for liver organ segmentation in CT images, which is called MSA-UNet. Our method makes a new design of U-Net encoder, decoder, skip connection, and context transition structure. These structures greatly enhance the feature extraction ability of encoder and the efficiency of decoder to recover spatial location information. We have designed many experiments on publicly available datasets to show the effectiveness of MSA-UNet. Compared with some other advanced segmentation methods, MSA-UNet finally achieved the best segmentation effect, reaching 98.00% dice similarity coefficient (DSC) and 96.08% intersection over union (IOU).

## Background

The liver, an important organ, undertakes the metabolic functions of the human body. The liver tumors will seriously threaten human lives and health. As mentioned in [1], liver cancer will become the sixth most common cancer and the fourth leading cause of cancer death in the world. Computed tomography (CT) is a commonly used diagnostic method in the liver lesions nowadays. CT images can reflect the shape, number, location, boundary and other information of liver tumors. Therefore, effective segmentation of liver tumor regions based on CT imaging technology has an important clinical value. Before setting the lesion area, it is very important to accurately describe the position of the liver. This process is usually manually marked by a professional radiologists. However, a large amount of image reading work is a serious burden for radiologists, and the final assessment results of different radiologists may be different due to subjective experience. Therefore, there is an urgent clinical need for an algorithm that can accurately and automatically segment the liver.

Recently, deep learning technology has shined in various computer vision tasks and achieved exciting results [2–7]. It was worth noting that image segmentation algorithms based on convolutional neural networks (CNNs) have achieved great success in many medical image segmentation tasks [2–4, 8, 9]. Compared with manual segmentation methods or traditional semi-automatic segmentation algorithms, CNNs have efficient feature extraction capability. It can perform fully automatic end-to-end training of data without too much empirical parameter settings or complex data pre-processing. In the field of image semantic segmentation, Long et al. [10] proposed fully convolutional networks (FCNs). FCNs contained a convolutional layer and a deconvolutional layer instead of a fully connected layer, which was different from the traditional image classification network [6, 7, 11, 12]. Therefore, the network can output segmented images with the same resolution size as the original image, thus solving the problem of image segmentation at the semantic level. Based on the FCNs, Ronneberger et al. [13] proposed U-Net segmentation network. The author designed a skip connection structure to transfer the feature map extracted by encoder to the corresponding network layer of decoder through cropping and copying. This allowed U-Net to obtain a more accurate pixel positioning which effected and segmented it in the cell wall. Inspired by [11], Li et al. [8] proposed a novel hybrid tightly connected U-Net—H-DenseUNet, which could be decomposed into 2D-DenseUNet and 3D-DenseUNet, the former can fully extract features in CT slices Information, the latter could effectively aggregate low-level features and high-level features. H-DenseUNet has been successfully applied in the segmentation task of healthy liver tissues and the liver tumor, but it was not easy to train and requires high experimental hardware environment. Milletari et al. [9] combined the idea of residual connection in [12] and proposed a method for 3D medical image segmentation—V-Net. Benefiting from the ability of residual connection to efficiently transfer feature information in the network layer, V-Net realized fast and accurate segmentation of prostate MRI images. In addition to introducing an effective convolution module in U-Net, it is worth considering how to reduce the semantic gap between encoder and decoder. Zhu et al. [14] proposed a new FCN by integrating U-Net and dilated dense network for hippocampal subfield segmentation. The method could avoid losing the detailed image information in the successive down-sampling steps, effectively fusing the low-level features with the high-level features. Zhou et al. [2] proposed UNet++ to solve the problem of excessive semantic gap between encoder and decoder. The author redesigned the skip connection structure in U-Net and introduced a dense convolution block, so that the skip connection can fuse the semantic information of different levels of encoder and pass it to decoder, significantly reducing the semantic gap between encoder and decoder. The residual connection was introduced between encoder and decoder. Instead of simply concatenating the feature maps from encoder to corresponding stage decoder, they were first passed through the convolutional layer chain with residual connections [3]. Then it was fused with the feature maps of decoder. Introducing the attention mechanism into the convolutional neural network structure was also popular [4, 5, 15, 16]. Attention Gates (AGs) [4] were added to the feature fusion of encoder and decoder in U-Net, allowing the model to learn to suppress irrelevant regions during training stage with only a small amount of parameters, while focusing on useful features information, improving the accuracy of the network to locate tissues and organs. From the perspective of channel and space, different attention mechanisms were designed [5]. The two attention mechanisms had their own focus points. Combining the two can improve the efficiency of solving semantic segmentation problems as a whole.

Based on the above research, we can find that the improvement of the convolution module in the original U-Net, the introduction of AGs or the redesign of skip connections and other methods can all improve the segmentation effect of U-Net to a certain extent. Inspired by the above-mentioned literature, our research combined the ideas of multi-scale convolution method and attention mechanism and proposed a method for liver organ segmentation in CT images—Multi-scale Attention U-Net (MSA-UNet). In general, the main contributions of this article are as follows:

1. Multi-scale Residual Block (MSRB) was designed. MSRB combined a multi-scale convolution module and residual connection to improve the feature extraction capability of the network. Multi-scale Attention Module (MSAM) was proposed, which could effectively strengthen useful features and suppress useless features. In order to make full use of the high-level semantic feature information between encoder and decoder, we added a structure called Attention Atrous Spatial Pyramid Pooling (AASPP) at the end of the encoder. In the skip connection, we designed the Residual Attention Skip Module (RASM) in order to effectively fuse the feature information and reduce the semantic gap between encoder and decoder.
2. Combining the U-Net structure with the designed improved module, the network structure MSA-UNet for liver organ segmentation in CT images was proposed.
3. Completed experiments on the proposed method on the public datasets. At the same time, some advanced semantic segmentation methods were selected for comparison, and all the experimental results were fully analyzed. The experimental results showed that our method obtained the best segmentation effect.

The rest of this article is organized as follows: In the second section, we first introduced the overview of MSA-UNet. Then we introduced in detail some of the new modules we proposed, including MSRB, MSAM, AASPP and RASM. In the third section, we declared the details of the datasets, the experimental environment, and the evaluation metrics used in the experiment. The fourth section is the result part. We first performed ablation experiments on some modules in MSA-UNet, and proved that each module had a certain effectiveness. Then we compared the proposed method with some advanced segmentation methods, including the comparison of different segmentation metrics and the analysis of the difference in training curves. Finally, with some discussion, we summarized the proposed new liver organ segmentation framework MSA-UNet in CT images.

## Methods

**Fig. 1:**
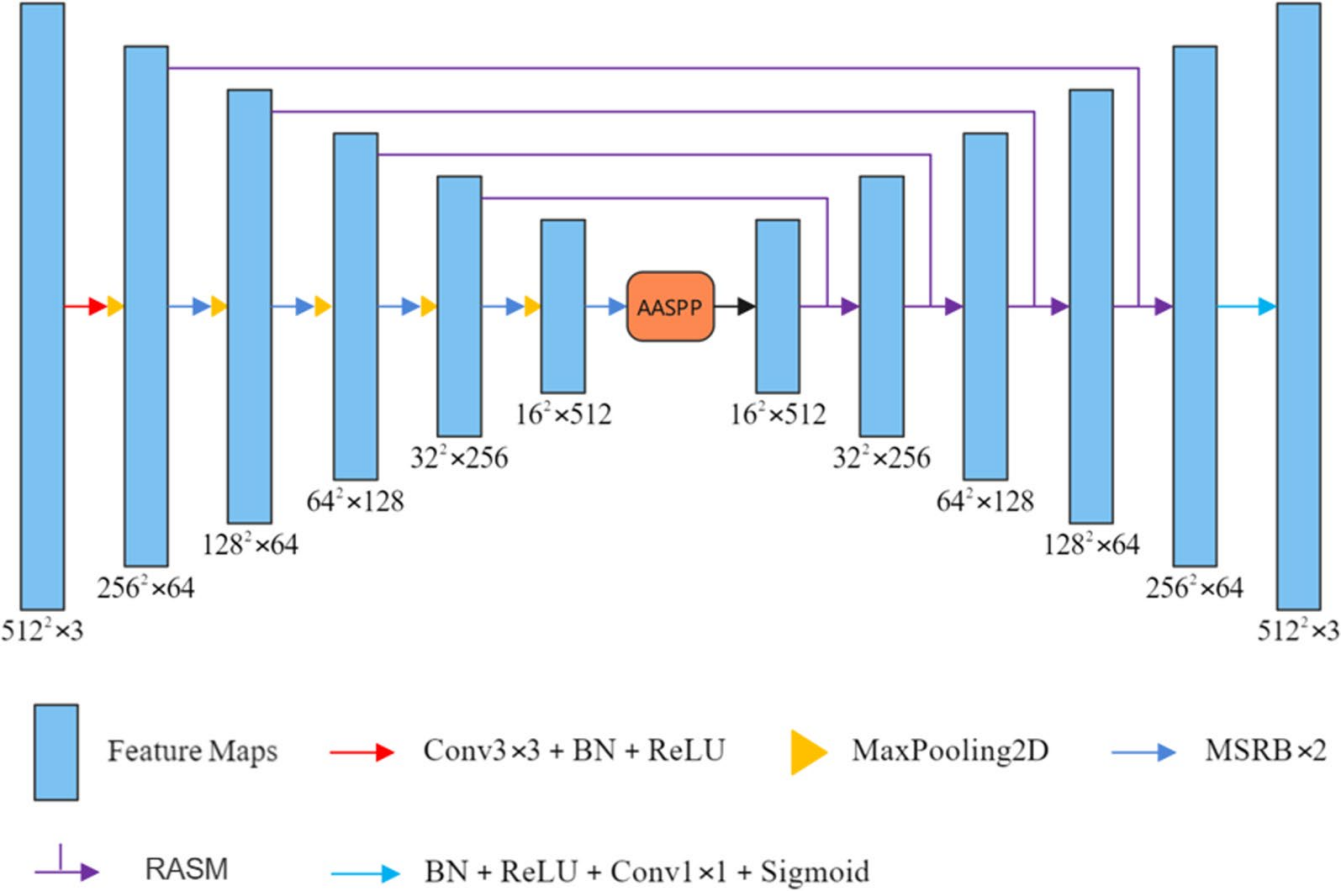
Overview of MSA-UNet architecture. In the encoder, we replaced the sequence of the two convolutional layers in the U-Net structure with the proposed MRSB. AASPP was a module used to capture contextual feature information, which was inserted between the encoder and the decoder. In addition, we did not use the skip connection in UNet, but used the proposed RASM. At the output of the model, we used a 1 × 1 convolution and a sigmoid activation function to obtain the final prediction result

In this work, based on the U-Net architecture, we combined it with the designed modules, and proposed a brand-new MSA-UNet model. The network architecture is shown in Fig. 1. MSA-UNet consists of four parts: encoder, decoder, context transition structure and skip connection. In the encoder, we used the proposed MSRB as the feature extraction block to improve the expressive ability of the convolutional network and extract richer deep features. AASPP was an improved structure of Atrous Spatial Pyramid Pooling (ASPP) [17]. We believe that embedding the attention module can further improve ASPP's ability of capturing contextual information, emphasizing useful features, and suppressing useless features. For decoders and skip connections, we proposed RASM to fuse low-level feature maps with high-level feature maps and perform further decoding through methods such as residual connections and attention mechanisms. Experiments have proved that the various modules proposed above have significantly improved the performance of U-Net and achieved convincing results in liver segmentation tasks. Additionally, we will introduce the new modules mentioned in MSA-UNet in detail, including MSRB, MSAM, AASPP, RASM.

### Multi-scale residual block

**Fig. 2:**
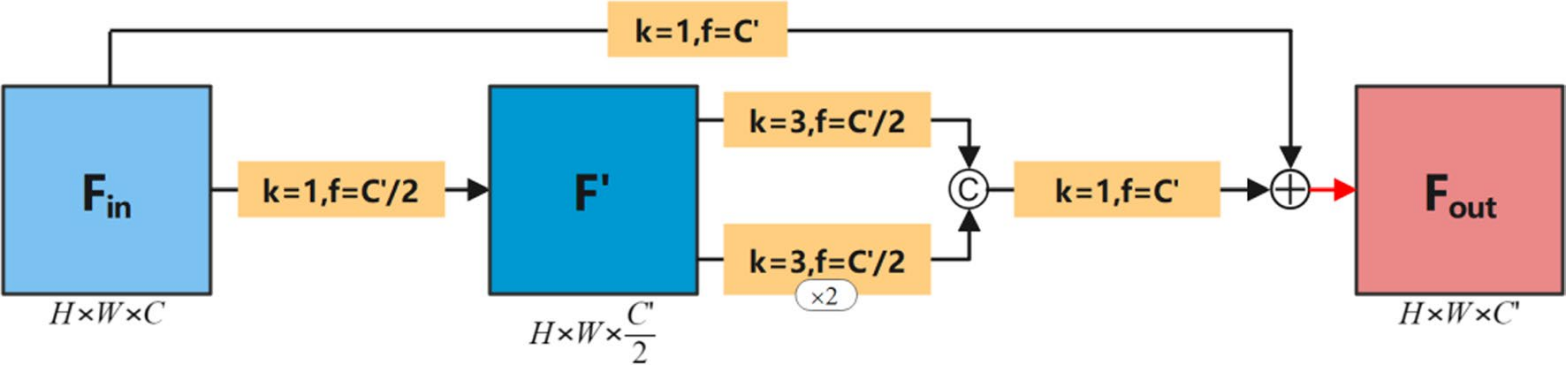
The proposed MSRB. Here, H, W, and C respectively represent the resolution height, width, and number of channels of the input feature maps. C′ represents the number of channels for output feature maps. The yellow square represents the convolution operation (where k represents the size of the convolution kernel, f represents the number of convolution kernels, and ×2 represents the convolution operation repeated twice), © represents the feature concatenation operation. $\oplus$ represents the point-by-point addition operation. The red arrow represents the DropBlock operation

CNNs is a method that can effectively extract the features of the input image. Simonyan et al. [6] proved that deepening the depth of the network layer can improve the final performance of the network. However, as the depth of the network layer deepens, the model will face problems such as over-fitting, gradient disappearance, and increase in computational complexity. These factors often makes it difficult to improve the performance of the model. Szegedy et al. [7] proposed a multi-scale convolution block called Inception module, which used convolution kernels of different scales to extract features and stitch each branch to aggregate the multi-scale features of the input image. Experiments showed that this move greatly improved the performance of the model. Nevertheless, the problem of gradient disappearance and explosion still existed. In response to this problem, He et al. [12] proposed the residual connection in 2016. Through the residual connection method, the efficiency of information dissemination within the network was significantly improved, and the number of parameters was reduced, and the phenomenon of gradient disappearance and explosion was also avoided. It was worth mentioning that [12] made the depth of CNNs breakthrough thousands of layers for the first time. Many of the network structures that emerged after this, such as [18, 19], have borrowed from the ideas of multi-scale convolution and residual connection, and have shown good performance in the speed and accuracy of the image classification task of ImageNet [20]. In the proposed MSA-UNet network, we used a multi-scale residual module (MSRB) in encoder part of the network. MSRB was mainly composed of two parallel convolution blocks. One branch is 3 × 3 convolution, and the other branch is composed of two 3 × 3 convolutions connected in series. Both of them are used to simulate the effect of 5 × 5 convolution to extract features of a larger receptive field. This was to prevent the direct use of a 5 × 5 convolution kernel, which would cause the parameter of the model to explode. In addition, outside the MSRB, there was a residual connection that added the features extracted from the multi-scale convolution block to the original features, which improved the efficiency of information dissemination within the network. It should be noted that each of the above convolutions was followed by operations such as batch normalization [21] and rectified linear unit (RELU) [22] activation. At the end of MSRB, we also added the DropBlock layer [23] to standardize the network. Due to the small size of the medical datasets, the model is likely to cause over-fitting during the training process. DropBlock [23] was a structured drop form, which could effectively prevent the over-fitting problem in convolutional networks, and has been successfully applied to computer vision tasks. Unlike Dropout [24], DropBlock discarded the continuous regions in the layer feature map instead of discarding independent random units. The final MSRB structure is shown in Fig. 2. We assumed that the input feature maps is $F_{in} \in R^{H \times W \times C}$, the output of MSRM $F_{out} \in R^{H \times W \times C^{\prime}}$ can be expressed by formula 1:

1 $$ F_{out} = d(f^{1} (F_{in} ) \oplus f^{1} [f^{3} (F_{in} );f^{3} (f^{3} (F_{in} ))]) $$

Here, $f^{k}$ represents the convolution operation with the kernel size $k \times k$. $d$ represents DropoutBlock operation. $\oplus$ means point-by-point addition operation, $[;]$ represents concatenation operation.

### Multi-scale attention module

**Fig. 3:**
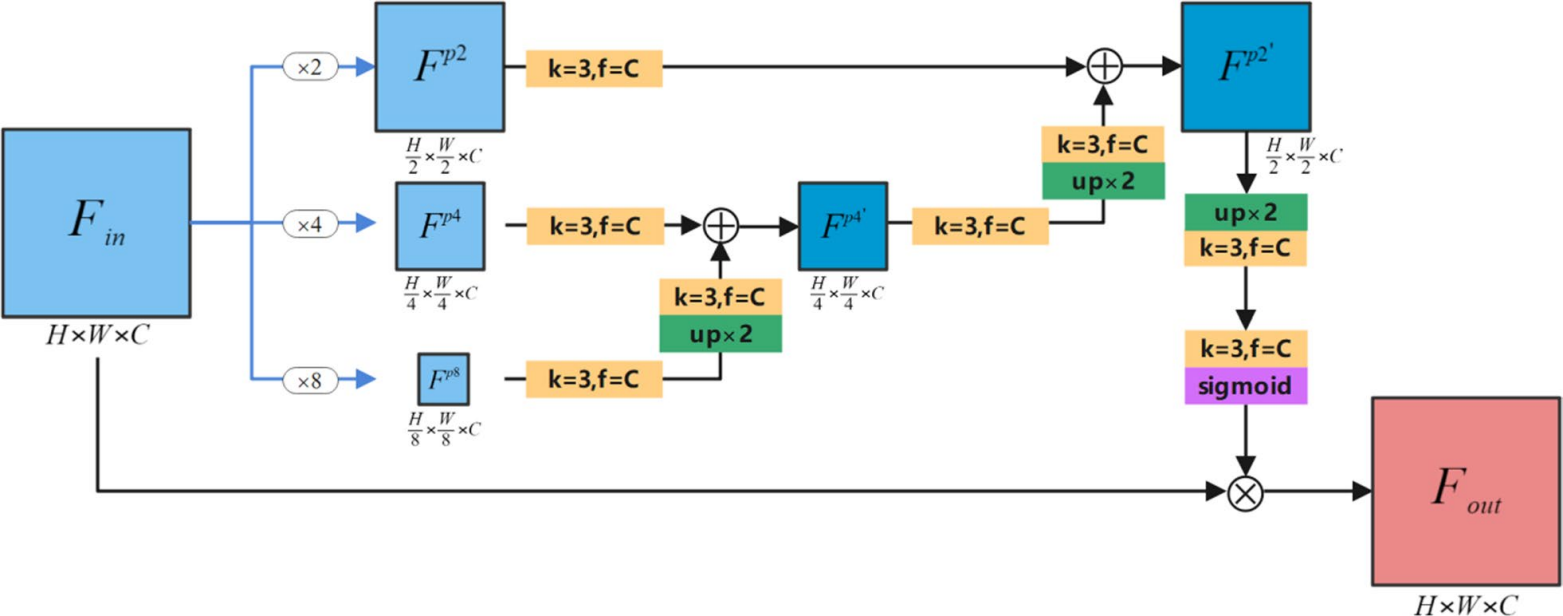
The proposed MSAM. Here, H, W, and C respectively represent the resolution height, width, and number of channels of the input feature maps. The yellow square represents the convolution operation (k represents the size of the convolution kernel, f represents the number of convolution kernels), and the green square represents the bi-linear up-sampling operation (×2 represents that the scale of the up-sampling is 2 times). $\otimes$ represents the point-by-point multiplication operation. $\oplus$ represents the point-by-point addition operation

Attention mechanism can improve the ability of networks to suppress useless information. It does not require significant changes to the network architecture and only needs to introduce a small number of parameters to obtain higher accuracy. Oktay et al. [4] introduced a soft attention mechanism and proposed the AGs module. The AGs module suppressed useless information in the input image through implicit learning of a trainable model, thereby highlighting salient features useful for specific tasks. Residual Attention Module (RAM) [25] was first proposed by Wang et al. and applied to image classification tasks. RAM actually used an hourglass structure (down-sampling n times, then up-sampling n times) to construct a soft attention mask, which significantly improved the accuracy of the image classification task. In this article, we designed a multi-scale attention module MSAM to optimize feature information. It was similar to RAM in which it compresses the input features first and then restored the shape, but the two were not the same. As shown in Fig. 3. MSAM first performed max-pooling of the input feature maps at different scales, then used convolution and bi-linear up-sampling methods to fuse multi-scale features stepwise, and finally generated feature attention maps through sigmoid function activation. We assumed that the input feature maps is $F_{in} \in R^{H \times W \times C}$. Firstly, $F_{in}$ is subjected to three different scale max-pooling operations of 2 × 2, 4 × 4, and 8 × 8 to obtain three feature maps of $F^{{p^{2} }}$, $F^{{p^{4} }}$ and $F^{{p^{8} }}$. Then $F^{{p^{4} }}$ and $F^{{p^{8} }}$ are added and fused by up-sampling and convolution to obtain the feature maps $F^{{{p^{4} }}^{{\prime}}}$. Similarly, $F^{{p^{2} }}$ and $F^{{{p^{4} }}^{{\prime}}}$ are fused in exactly the same way to obtain the feature maps $F^{{{p^{2} }}^{{\prime}}}$. Finally, use up-sampling, convolution, sigmoid function activation and other methods to obtain the final feature attention maps, and multiply it with the input $F_{in}$, and finally obtained output feature maps $F_{out} \in R^{{H \times W \times C^{{}} }}$. It was worth mentioning that the size of the feature attention maps obtained by our proposed MSAM was the same as the input $F_{in}$. In this way, the attention mechanism we proposed could perform more comprehensive attention weight distribution from the two dimensions of channel and space. The process of MSAM can be expressed by formula 2:

2 $$ F_{out} = F_{in} \otimes \sigma (f^{1} (b^{2} (f^{3} (F_{{}}^{{p2^{{\prime}} }} )))) $$

Here, $F^{{{p^{2} }}^{{\prime}}}$ represents the attention map mentioned above. $f^{k}$ represents the convolution operation with the kernel size $k \times k$. $b^{k}$ represents a bi-linear interpolation up-sampling operation with an up-sampling scale of $k \times k$. $\otimes$ represents the point-by-point multiplication operation. $\sigma$ represents sigmoid activation function.

### Attention atrous spatial pyramid pooling module

**Fig. 4:**
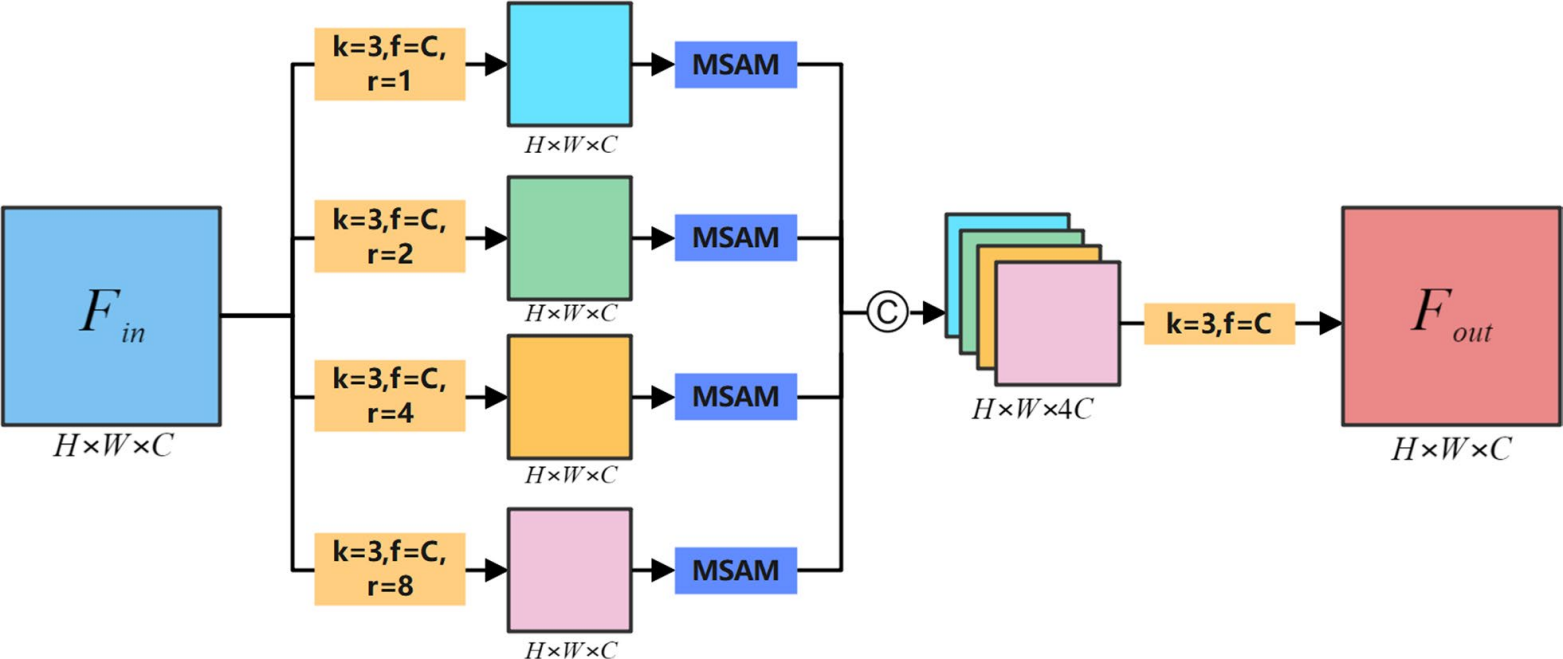
The proposed AASPP. Here, H, W, and C respectively represent the resolution height, width, and number of channels of the input feature maps. The yellow square represents the convolution operation (k represents the size of the convolution kernel, f represents the number of convolution kernels, and r represents the dilated factor). © represents the feature concatenate operation

The context transition structure between encoder and decoder plays a crucial role in the overall performance of the model. The PPM [26] structure was proposed to capture contextual information. PPM used a multi-scale pooling operation to aggregate the input feature maps, and then re-fuse the features through convolution and up-sampling methods. Different from [17, 26] used ASPP to extract contextual information. ASPP performed multi-scale feature extraction on the input feature maps through dilated convolution operations with different dilated factor, and then fused the final multi-scale features to output. Compared with the pooling operation, the dilated convolution could extract multi-scale features without changing the spatial resolution of the feature map. And the obtained features could be directly fused without the need for subsequent supplementary up-sampling operations to restore the dimensionality as in [26], because this may require additional training time and memory. Therefore, inspired by [17], we selected ASPP as the basic structure for capturing contextual information and proposed the AASPP structure. As shown in Fig. 4, AASPP combined the attention mechanism with ASPP and optimized the feature maps obtained in ASPP. We assumed that the input feature map is $F_{in} \in R^{H \times W \times C}$, we designed four parallel branches to extract features of $F_{in}$, and each branch was composed of dilated convolutions with different dilated factor. And after each branch, MSAM was added to optimize the features. Finally, we used the concatenation operation to fuse the feature maps obtained from the four branches, and then obtained the output $F_{out} \in R^{{H \times W \times C^{{}} }}$ through 1X1 convolution. The process of AASPP can be expressed by formula 3:

3 $$ F_{out} = f_{1}^{1} [\alpha (f_{1}^{1} (F_{in} ));\alpha (f_{2}^{3} (F_{in} ));\alpha (f_{4}^{3} (F_{in} ));\alpha (f_{8}^{3} (F_{in} ))] $$

Here, $f_{r}^{k}$ represents a dilated convolution operation with a dilated factor of $r$ and kernel size of $k \times k$. $\alpha$ represents the MSAM attention mechanism. $[;]$ represents concatenation operation.

### Res attention skip module

In U-Net, the role of skip connection was to directly connect the feature mapping between encoder and decoder. However, the features obtained by the encoder are calculated early in the network and contain less semantic information, which we called low-level features. On the other hand, the features of the decoder input the information obtained by the deep calculation of the network, which we called high-level features.

**Fig. 5:**
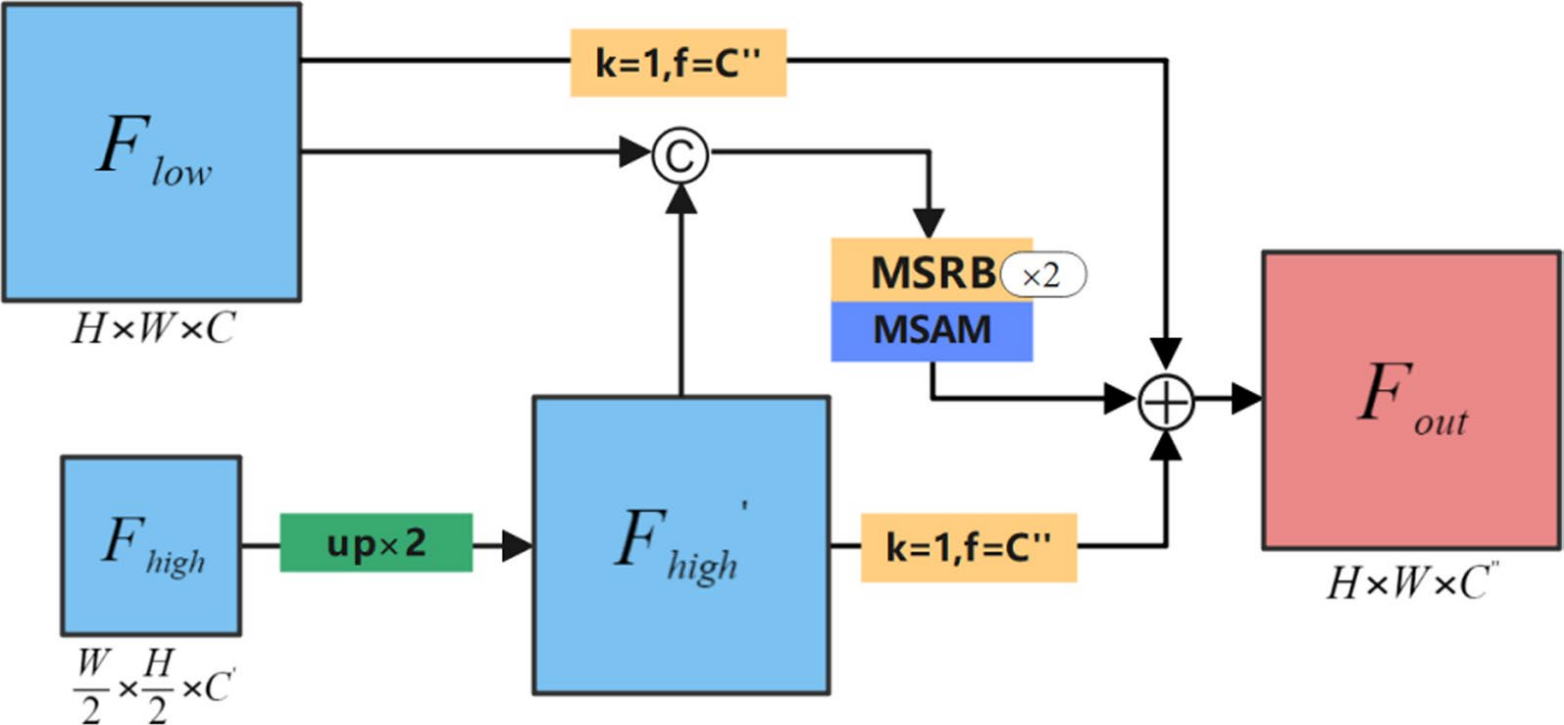
The proposed RASM. Here, H, W, and C respectively represent the resolution height, width, and number of channels of the input low-level feature maps. C′ represents the number of channels for output feature maps. C″ represents the number of channels for output feature maps. The yellow square represents the convolution operation (where k represents the size of the convolution kernel, f represents the number of convolution kernels), and © represents the feature concatenation operation. ⊕ represents the feature addition operation

Obviously, there is a certain semantic gap between low-level features and high-level features, and directly connecting the two may adversely affect the prediction results. In response to this problem, Ibtehaz et al. [3] added convolutional layers and residual connections to skip connections, and proposed ResPath to reduce the semantic gap between low-level features and high-level features. Szegedy et al. [18] proposed the GAU structure, letting high-level features containing rich semantic information used the global information provided by global pooling as a guide to select low-level features. It can be seen that adding residual connections to the skip connection structure and the improvement of the attention mechanism were effective. Inspired by the above-mentioned literature, we proposed RASM for fusion of high-level features and low-level features. We assumed that the input low-level feature maps is $F_{low} \in R^{H \times W \times C}$, and the high-level feature maps is $F_{high} \in R^{{\frac{H}{2} \times \frac{W}{2} \times C^{{\prime}} }}$. Specifically, we first used bi-linear interpolation to up-sample $F_{high}$ twice to generate $F_{high}^{{\prime}}$. At this time, the resolution of $F_{high}^{{\prime}}$ is consistent with the low-level features. Then we concatenated $F_{high}^{{\prime}}$ and $F_{low}$, and after two MSRB operations, and finally through MSAM to optimize its feature information. Externally, we designed two residual connections to fuse all feature maps and finally get the output $F_{out} \in R^{{H \times W \times C^{{\prime\prime}} }}$. Figure 5 shows the overall structure of RASM. The process of RASM can be expressed by formula 4:

4 $$ F_{out} = f^{1} (F_{low} ) \oplus f^{1} (b^{2} (F_{high} )) \oplus \alpha (\theta^{2} [b^{2} (F_{high} );F_{low} ]) $$

Here, $f^{k}$ represents the convolution operation with kernel size $k \times k$. $b^{k}$ represents a bi-linear interpolation up-sampling operation with an up-sampling scale of $k \times k$. $\theta^{k}$ means that the MSRB operation is executed $k$ times in sequence. $\alpha$ represents the MSAM attention mechanism. $[;]$ represents concatenation operation. $\oplus$ represents the point-by-point addition operation.

### Loss function

The binary cross entropy loss function is a loss function used in classic image binary classification tasks. In this experiment, the segmentation task of the liver can also be regarded as a pixel-level binary classification task that distinguishes the liver from the background. Therefore, we selected the binary cross-entropy loss function as part of the loss function used in the experiment. $L_{bce}$ can be defined by formula 5.

5 $$ L_{bce} = - y\log \left( p \right) - \left( {1 - y} \right)\log \left( {1 - p} \right) $$

where y represents the ground truth value of the pixel in the image, and p represents the result of the algorithm prediction.

However, simply using the binary cross-entropy loss function may be difficult to handle complex medical image segmentation tasks. Therefore, we combined the use of the dice loss function [9]. The dice loss function can effectively deal with the imbalance of the number of categories in the medical image segmentation problem, and it can improve the training performance of the network. Formula 6 shows the calculation method of $L_{dcl}$.

6 $$ L_{dcl} \left( {y,p} \right) = 1 - \frac{{2\sum\nolimits_{i = 1}^{N} {p_{i} y_{i} } }}{{\sum\nolimits_{i = 1}^{N} {y_{i} + \sum\nolimits_{i = 1}^{N} {p_{i} } } }} $$

Here, N represents the number of all pixels on the input image, $y_{i}$ represents the ground truth value of pixel i, $y_{i} \in \left\{ {0,1} \right\}$. $p_{i}$ represents the algorithm prediction of pixel i, $p_{i} \in \left( {0,1} \right)$.

Finally, the total loss function used in this experiment is defined as:

7 $$ L_{total} = \alpha L_{bce} + \beta L_{dcl} $$

Here, α and β are the weight coefficients used to balance the two loss functions. We tested various values of α and β in the training phase, analyzed the training curve and selected the best coefficients. Finally, we selected α = 0.5 and β = 1.0 as the final weight coefficients used in this experiment.

## Experiment

### Dataset and pre-processing

We mixed 3Dircadb01 [27] and MICCAI-Sliver07 [28] datasets for the experiments. Both data can be applied for and obtained on public websites. Sliver07 contains 20 CT data with liver labels. The number of slices contained in each CT varies from 64 to 512, and the slice thickness varies from 0.5 to 5.0 mm. 3Dircadb01 includes 20 intravenous phase enhanced CT volumes from different European hospitals using different CT scanners. These data are provided in dicom format and are accompanied by marked images corresponding to each region of interest. The resolution of all CT slices in 3Dircadb01 is 512 × 512, and the number of slices in each sample varies from 74 to 260.

In the process of data pre-processing, we first truncated the CT values to the range of [− 200, 250] to remove irrelevant tissues. On the other hand, due to the characteristics of CT scanning imaging, the boundary between soft tissues with similar density is not clear, we used contrast-limited adaptive histogram equalization (CLAHE) [29] to increase the contrast between different tissues. We selected the CT image resolution of the input model to 512 × 512, and divided all CT data into training set, validation set and test set according to the proportion of 8:1:1. In the training phase, in order to expand the datasets to prevent over-fitting, we also used data augmentation methods such as random horizontal and vertical flipping, random clipping of the image (the clipping ratio is at most 10% of the original image), random translation (xy axis direction ± 10%) and so on.

### Implementation details

The algorithm model proposed in this paper was built by Keras [30] (using tensorFlow backend), convolution kernel was set to the initializer method proposed by [31]. The optimizer used in the model was the Adam optimizer [32]. The initial learning rate was set to 0.001, the epoch was set to 300, and the training batch size was set to 6. It was worth mentioning that in the training phase, we always monitor the loss changes of the model on the verification set to make different decisions on the learning rate and other parameters. Specifically, if the loss of the verification set did not decrease for 8 epoch, the learning rate would be reduced to half of the current value. If the loss of the verification set did not decrease for 20 epoch, The training process needed to stop in advance to prevent over-fitting. Finally, the output of the network consisted of the probability map of the background and foreground. We selected the pixel value whose probability is higher than 0.5 as the liver region and the rest as the non-liver region. In addition, we also made a simple data augmentation to the test set (including horizontal flip, vertical flip, etc.), and averaged the results of the model on these enhanced data as the final prediction results. The hardware environment of this experiment is 8 GB Intel i7-9700K, NVIDIA GeForce RTX 2080ti.

### Evaluation metrics

We used a total of four objective and general segmentation model evaluation metrics to evaluate the difference between the model prediction results and the ground truth. It includes dice similarity coefficient (DSC), intersection over union (IOU), recall and precision. DSC and IOU can be used to evaluate the overall difference between the model prediction results and the ground truth. Recall is an important reference index in clinical practice. Precision is usually used to evaluate the overall quality of the segmentation results. The formulas for these evaluation metrics can be expressed as follows:

8 $$ DSC(G,P) = \frac{{2\left| {G \cap P} \right|}}{\left| G \right| + \left| P \right|} $$

9 $$ IOU(G,P) = \frac{{\left| {G \cap P} \right|}}{{\left| {G \cup P} \right|}} $$

10 $$ precision = \frac{{\left| {G \cap P} \right|}}{\left| P \right|} $$

11 $$ recall = \frac{{\left| {G \cap P} \right|}}{\left| G \right|} $$

Here, G and P respectively represents the ground truth and the model prediction results. For DSC and IOU, the range is 0 to 1, 0 means no overlap, 1 means perfect segmentation. The larger the values of these four indicators, the larger the overlapping area between the model prediction results and the ground truth, the higher the similarity, and the greater the accuracy of the segmentation.

## Results

In this section, we compared MSA-UNet with other advanced segmentation methods. We selected U-Net as the baseline model of the experiment, and selected three advanced network structures such as CE-Net [33], UNet++ [2] and Deeplabv3+ [34] as the comparative experimental model. It was worth mentioning that for the sake of fairness, we trained each model under the same experimental conditions (loss function, learning rate, optimizer, etc.). We saved models that perform best on the validation set and evaluate them on the test set. Finally, the experimental results showed that MSA-UNet achieved better segmentation performance, and the performance on the test set was better than other comparison models.

### Comparative analysis between different models

**Fig. 6:**
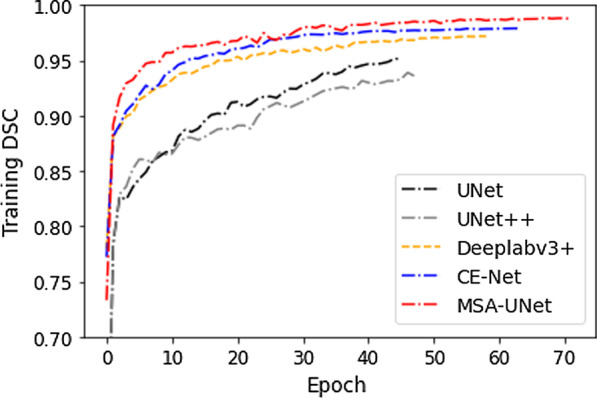
The increase in the accuracy of different models in the training process

Firstly, we analyzed the learning process of different segmentation methods. Figure 6 shows the increase in the accuracy of different models in the training process. As shown in the Fig. 6, MSA-UNet finally achieved the best effect on the training set, with the highest DICE of 98.00%, IOU of 96.08%, Precision of 97.17% and Recall of 98.85%. At the same time, the accuracy of U-Net model was the lowest after convergence, with the DICE of 89.23%, IOU of 80.93%, Precision of 92.97% and Recall of 86.49%, and the training process was stopped in 46 epoch. UNet++ was better than U-Net because of its internally redesigned skip connection structure, but it also stopped the training process in 48 epoch. On the other hand, due to the introduction of improved structures such as residual convolution block and ASPP, the performance of CE-Net and Deeplabv3+ was improved accordingly, and their performance surpassed that of U-Net and UNet++, to complete the convergence process in 64 and 59 epoch, respectively.

Table 1 shows the performance of each model on the test set. We used four evaluation metrics to measure the accuracy of the segmentation results, DICE similarity coefficient (DSC), intersection over union (IOU), recall and precision. We predicted and calculated the evaluation metrics for each test sample and finally obtained the mean value and standard deviation of the evaluation metrics of the model on test set. We presented these results in Table 1. As can be seen from Table 1, our method achieved better results in liver segmentation than other models, achieving the IOU increased 15.14%, the DSC increased 8.76%, the precision increased 4.20%, and the recall increased 12.36% over U-Net. In all comparison models, CE-Net and Deeplabv3+ perform better, and the DSC reached 97.67% and 96.94%, respectively. Our method was still better than all comparison models, and the DSC reached the highest level of 98.00%.

**Table 1:** Segmentation results of methods on the test set

Method	DSC [%]	IOU [%]	Precision [%]	Recall [%]
UNet [13]	89.23 ± 4.98	80.93 ± 8.12	92.97 ± 3.87	86.49 ± 9.59
UNet++ [2]	92.10 ± 3.62	85.56 ± 6.04	93.49 ± 4.96	91.23 ± 6.34
DeepLabv3+ [34]	96.94 ± 2.85	94.19 ± 4.45	95.42 ± 4.37	98.63 ± 1.22
CE-Net [33]	97.67 ± 0.81	95.46 ± 1.53	96.76 ± 1.45	98.61 ± 1.22
MSA-UNet	**98.00** ± **0.38**	**96.08** ± **0.74**	**97.17** ± **0.85**	**98.85** ± **0.70**

The best performance evaluation metrics are in bold

**Fig. 7:**
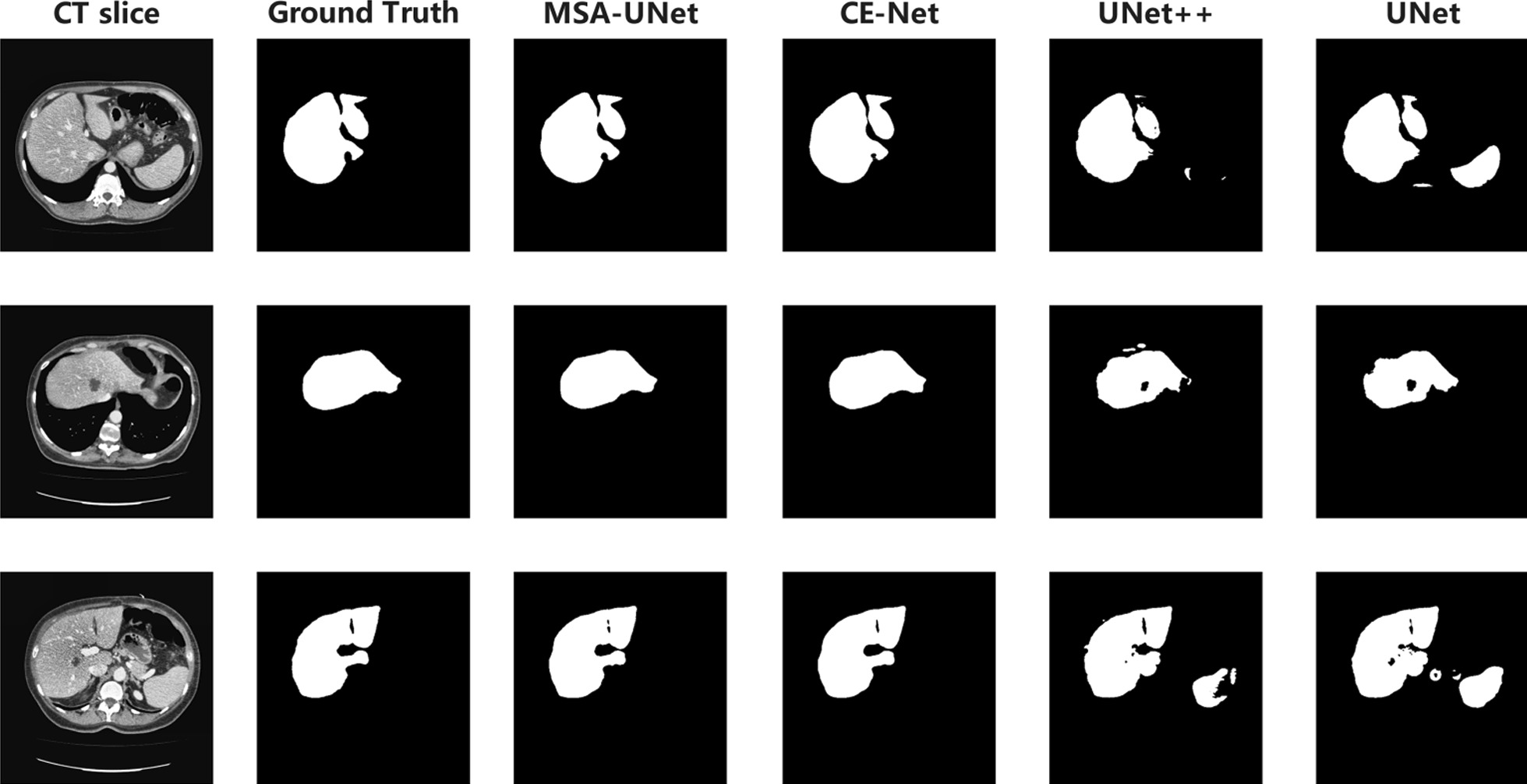
Examples of the segmentation results of different methods on the test set

Organ and tissue segmentation with blurred boundaries was a difficult task in medical image segmentation. We listed some randomly selected samples of segmentation results. Figure 7 shows an example of the predicted results of the model. We could observe that the prediction image of the MSA-UNet model could retain more accurate liver boundary information and achieved a more perfect prediction effect than other models. The above experimental results verified the superiority of our proposed method compared with other methods.

### Ablation analysis of the MSA-UNet

**Fig. 8:**
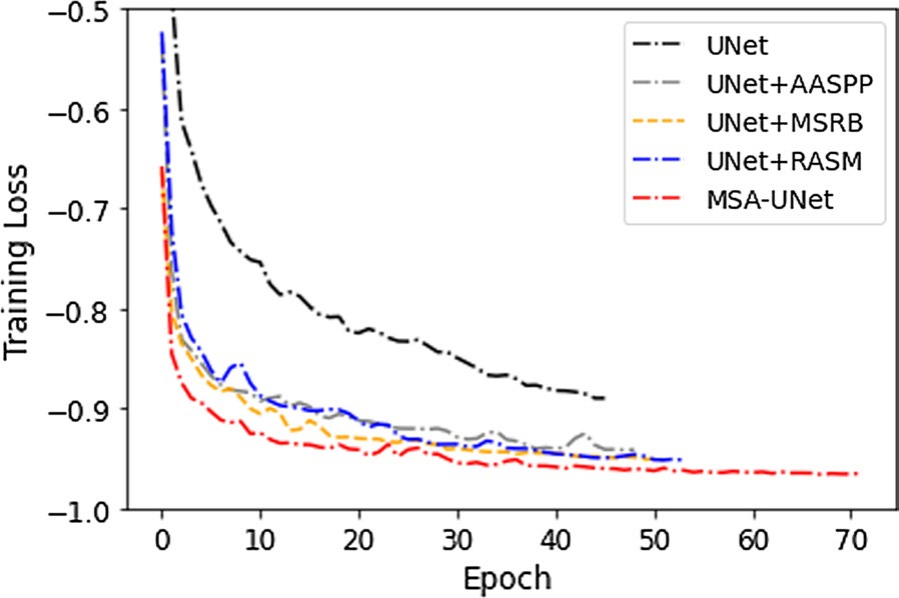
The decrease in the loss of different models in the training process

In this part, we used ablation analysis to prove the effectiveness of each component in the MSA-UNet network. Similarly, we selected U-Net as the baseline model of the experiment, and added modules such as RASM, MSRB, AASPP on the basis of U-Net as the comparison models. During the training phase, we recorded the loss changes of each model to monitor the performance of the model and presented the final results in Fig. 8. As shown in the Fig. 8, modules such as RASM, MSRB, AASPP could significantly improve the segmentation effect of U-Net and made it converge to a lower loss value in the training process. The ablation experiment proved the effectiveness of our proposed module. It was worth noting that the loss value of MSA-UNet converged to a minimum, which proved the effectiveness of combination of multiple modules.

Table 2 compares the performance of U-Net, MSA-UNet and three ablation experimental models on the test set. It could be found that the final MSA-UNet was the best among all the selected evaluation metrics. At the same time, our proposed modules such as MSRB, AASPP, RASM improved the performance of U-Net to some extent, which showed the effectiveness of each module.

**Table 2:** Segmentation results of ablation methods on the test set

Method	DSC [%]	IOU [%]	Precision [%]	Recall [%]
UNet	89.23 ± 4.98	80.93 ± 8.12	92.97 ± 3.87	86.49 ± 9.59
UNet + MSRB	97.10 ± 1.02	94.38 ± 1.91	95.76 ± 1.83	98.52 ± 1.51
UNet + AASPP	96.83 ± 2.35	93.95 ± 4.09	96.21 ± 2.81	97.59 ± 3.55
UNet + RASM	97.13 ± 1.07	94.45 ± 1.98	95.60 ± 2.16	98.75 ± 1.18
MSA-UNet	**98.00 ± 0.38**	**96.08 ± 0.74**	**97.17 ± 0.85**	**98.85 ± 0.70**

The best performance evaluation metrics are in bold

## Discussion

This study proposed a solution to the problem of liver segmentation in CT images. Based on U-Net, combined with multi-scale idea and attention mechanism, we designed a new segmentation model. We tested this method on the public datasets, calculated the segmentation metrics and compared several advanced semantic segmentation methods. The experimental results showed that our method achieved the best effect on each metrics. The experimental training curve showed that there was no over-fitting phenomenon in our model on small datasets. We also designed ablation experiments to prove the effectiveness of the proposed module, and combined the proposed innovation module with U-Net respectively to analyze its effectiveness. The results of ablation experiments showed that each module we proposed can improve the performance of U-Net to some extent. The above experimental results showed that our method had some advantages in automatic liver segmentation, but there was still some room for improvement. We planned to use a large number of sample data from affiliated hospitals to conduct more comprehensive experiments in the future. On the other hand, we planned to lightweight the whole network to reduce the number of parameters of the model without losing accuracy, so as to better assist the automatic, fast and efficient segmentation of the liver.

## Conclusion

A network structure, MSA-UNet, which was suitable for liver organ segmentation in CT images, was proposed. On the basis of U-Net, we combined the ideas of multi-scale convolution module and attention mechanism to design a variety of innovative structures to improve its performance. At the same time, we mixed the binary cross-entropy loss function and the dice loss function in order to alleviate the imbalance between foreground and background pixels in medical image segmentation. The experimental results on the open datasets showed that our proposed method is feasible and effective. Compared with some advanced segmentation algorithms, our method achieved the best segmentation effect, and was better than four advanced segmentation architectures in segmentation metrics such as DSC, IOU, recall and precision. MSA-UNet has proposed a substantial improvement for automatic segmentation of liver organs in CT images, and was expected to further become a clinical auxiliary tool for liver organ segmentation in the future.

## Data Availability

All datasets participating in this study can be found on this website https://www.ircad.fr/research/3dircadb/ and https://zenodo.org/record/2597908#.YURZz44zZPY. The validation set and the test set that support the conclusions of this article can be accessed by making a request to the corresponding author.

## Abbreviations

CNNs: Convolutional neural networks
FCNs: Fully convolutional networks
ASPP: Atrous spatial pyramid pooling
PPM: Pyramid pooling module
AGs: Attention gates
